# Michigan

**Published:** 1896-05

**Authors:** 


*Michigan.* The Jersey Cattle-breeders' Association at their meeting in Lansing, after discussing the subject of tuberculosis, gave forth the opinion that many valuable animals had been destroyed on the tuberculin-test, and destroyed without finding the presence of tuberculosis. We would like to know where, when, and what percentage they claim for these unusual statements?

